# Impact of Maternal Smoking on Obstetric and Neonatal Outcomes in Twin Pregnancies: A Narrative Review

**DOI:** 10.3390/jcm13237329

**Published:** 2024-12-02

**Authors:** Cristina Juliá-Burchés, Alicia Martínez-Varea, José Morales-Roselló, Vicente Diago-Almela

**Affiliations:** 1 Department of Obstetrics and Gynecology, La Fe University and Polytechnic Hospital, 46026 Valencia, Spain; julia_cribur@gva.es (C.J.-B.); jose.morales@uv.es (J.M.-R.); diago_vicalm@gva.es (V.D.-A.); 2 Department of Medicine, CEU Cardenal Herrera University, 12006 Castellón de la Plana, Spain; 3 Faculty of Health Sciences, Universidad Internacional de Valencia, 46026 Valencia, Spain; 4 Department of Pediatrics, Obstetrics and Gynecology, Faculty of Medicine, University of Valencia, 12006 Valencia, Spain

**Keywords:** maternal smoking, tobacco exposure, twin pregnancies, multiple gestations, obstetric outcomes, neonatal outcomes, preterm birth, fetal growth restriction (FGR), preterm premature rupture of membranes (PPROM), fetal death, respiratory disorders, smoking cessation, placental insufficiency

## Abstract

Maternal smoking, including both traditional cigarettes and electronic ones, is a significant modifiable risk factor associated with adverse perinatal outcomes, especially in twin pregnancies. This narrative review aims to explore the impact of maternal smoking on obstetric and neonatal outcomes in twin pregnancies, which inherently carry a higher risk of complications. A literature search was conducted using the PubMed and EMBASE databases, selecting studies published between January 1994 and October 2024. The findings demonstrate a clear association between smoking and increased risks of preterm birth and fetal growth restriction (FGR) in twin pregnancies. These risks are exacerbated when smoking is combined with other factors, such as preeclampsia and elevated body mass index (BMI). Smoking was also associated with long-term post-natal complications, including respiratory problems like asthma, as well as cognitive and behavioral disorders. However, an association with preeclampsia was not found, and further studies are needed to clarify the relationship in the fields of preterm premature rupture of membranes (PPROM) and fetal death. The adverse effects of smoking are primarily due to reduced oxygen supply to the fetus, caused by nicotine-induced vasoconstriction and carbon monoxide exposure, leading to placental insufficiency and fetal hypoxia. These effects are amplified in twin pregnancies due to the increased physiological demands. The review highlights that smoking cessation interventions during pregnancy are crucial to mitigate these risks and improve maternal and neonatal health outcomes.

## 1. Introduction

Maternal smoking, including both traditional cigarettes and electronic ones, is one of the main modifiable risk factors associated with adverse perinatal outcomes [1]. Therefore, detection and intervention for smoking cessation during pregnancy is an essential tool for achieving the discontinuation of this habit to prevent gestational complications [2]. What is more, pregnancy also offers an opportunity to educate the partner and other family members of the pregnant woman about the benefits of quitting smoking for themselves, the woman, and the future newborn [3]. Hence, all pregnant women should be asked about their smoking habits, including whether they have ever smoked, whether they did while pregnant, and whether they are currently smoking, emphasizing the number of cigarettes smoked per day [2,3].

In recent years, the increasing efforts in public health education have contributed to a general decline in smoking rates, although many women continue to smoke during pregnancy [1]. The true prevalence of smoking during pregnancy is challenging to determine, not only due to incomplete records but also because most studies rely on self-reported smoking habits, which are, therefore, subject to underreporting of actual cases [4]. In fact, studies using biochemical markers, including carbon monoxide (CO) and urinary cotinine, have shown that pregnant women do not always disclose their active smoking status [4]. Moreover, passive tobacco exposure can result in detectable levels of CO and cotinine in blood, saliva, urine, and exhaled air; however, the levels are typically lower than in active smokers [4].

Several mechanisms have been proposed to explain the adverse pregnancy outcomes associated with maternal smoking. These include impaired fetal oxygenation, as well as exposure to toxins [5]. On the one hand, smoking during pregnancy causes a reduction in the oxygen supply to the fetus through several pathways. The release of CO leads to the formation of carboxyhemoglobin, a protein formed by the binding of CO to hemoglobin, with multiple effects on systemic and fetal oxygen supply [6]. Carboxyhemoglobin is slowly eliminated from fetal circulation, reducing tissue oxygenation by competitively inhibiting oxyhemoglobin and causing a leftward shift in the oxyhemoglobin dissociation curve [6]. Additionally, smoking-induced oxidative stress may increase placental production of mitochondrial reactive oxygen species, such as nitric oxide, which reacts with superoxide radicals to produce peroxynitrite, damaging placental function [7]. Indeed, studies of the placentas of smoking patients have shown structural changes, including a reduction in capillary volume fraction and an increase in chorionic villous thickness compared to non-smokers [7]. Both factors may contribute to abnormal placental gas exchange [6,7]. On the other hand, nicotine acutely decreases intervillous perfusion, possibly due to the vasospasm it induces, thereby increasing vascular resistance and reducing blood flow through the uterine arteries [7].

All of this results in obstetric complications such as intrauterine growth restriction, increased risk of preeclampsia, placental abruption, preterm birth, premature rupture of membranes, and fetal/neonatal death [8,9,10]. Furthermore, it also increases the risk of miscarriage, ectopic pregnancy, and placenta previa [8,9,10]. Regarding the postnatal period, smoking during pregnancy may predispose offspring to a higher risk of behavioral and cognitive disorders, overweight and obesity, respiratory disorders, and tobacco addiction [1]. Therefore, tobacco negatively affects both maternal and fetal health, with neonatal consequences that can also be long-term [1,8,9,10].

Twin pregnancies are associated with higher rates of almost all potential gestational complications, with the only exceptions being post-term pregnancy and macrosomia [11]. The most frequent risks are fetal growth restriction and preterm birth [11]. The latter is the most severe complication, both because of its frequency and its implication in perinatal morbidity and mortality, as well as its medium and long-term impact [11].

Therefore, considering that the primary adverse gestational and neonatal outcomes related to smoking have also been demonstrated in twin pregnancies, it would be advisable to assess the impact of smoking tobacco in these pregnancies, which already have an inherently higher risk of adverse outcomes.

## 2. Materials and Methods

### 2.1. Search Strategy and Selection Criteria

A literature review was conducted by searching for published studies in the PubMed and EMBASE databases written in Spanish or English from January 1994 to 4 October 2024. The search terms used were tobacco AND twin pregnancies, smoking AND twin pregnancies, and nicotine AND twin pregnancies. To systematically structure the search and selection of studies, the PICO approach (Patient/Problem, Intervention, Comparator, and Outcome) was applied, allowing for a clear definition of the inclusion criteria and expected outcomes. This approach facilitated the identification of relevant studies addressing the impact of smoking on twin pregnancies and its obstetric and neonatal consequences.

Hence, the applied inclusion criteria were: (1) retrospective or prospective studies that provided a detailed description of the methodological approach used, including cohort studies, case-control studies, case reports, case series, narrative reviews, literature reviews, and meta-analyses, (2) studies with full-text available, (3) studies written in Spanish or English from January 1994 to 4 October 2024, (4) studies that addressed the maternal and neonatal impact of tobacco in twin pregnancies or the postnatal effects in the newborns. The following studies were excluded: (1) studies that did not provide a detailed description of the methodology used, as well as the number of patients or studies included, (2) studies with no full text available, (3) studies in written languages other than Spanish or English, and (4) those irrelevant to the subject of the narrative review after reading the title and the abstract. Additionally, the bibliography of the papers selected was screened in the search for other studies that accomplished the criteria mentioned above to be included.

To ensure methodological rigor, the quality of the observational studies was assessed using the Newcastle–Ottawa Scale (NOS), which evaluates three main domains: participant selection, comparability of study groups, and outcome assessment. Only studies scoring in the high-quality range (7–9 points) were included in the review. The AMSTAR-2 tool was used to evaluate methodological quality for reviews and meta-analyses, and only high-quality studies were included. This approach ensured that the included studies demonstrated robust methodologies and minimized the risk of bias.

### 2.2. Data Extraction

The main objective of this review was to compare the maternal and fetal gestational outcomes in patients with twin pregnancies exposed to tobacco, both actively and passively, with those who were non-smokers. The secondary objectives of the study were, firstly, to evaluate the physiopathology involved in the process; secondly, to compare the results with single pregnancies; and, thirdly, to examine the long-term neonatal complications.

The selected studies were analyzed for the following data: (1) basic information about the studies, such as publication year, first author, and study design; (2) characteristics of the study population, including the type of participants, sample size, and location; (3) type of smokers: active or passive; and (4) results and conclusions of the studies. The essential characteristics of the included studies are summarized in Table 1.

### 2.3. Data Synthesis

A narrative review was conducted using the data gathered from the selected studies. ZOTERO was employed to sort the articles and remove any duplicates.

The adverse gestational and perinatal outcomes were analyzed by examining the potential complications described in the literature. The different pathophysiological hypotheses proposed were examined individually, thoroughly assessing their plausibility. Extensive research was conducted to compare the results with singleton pregnancies. Finally, post-neonatal complications were addressed by analyzing the reported long-term complications in newborns.

## 3. Results

### 3.1. Included Articles

**Figure 1:**
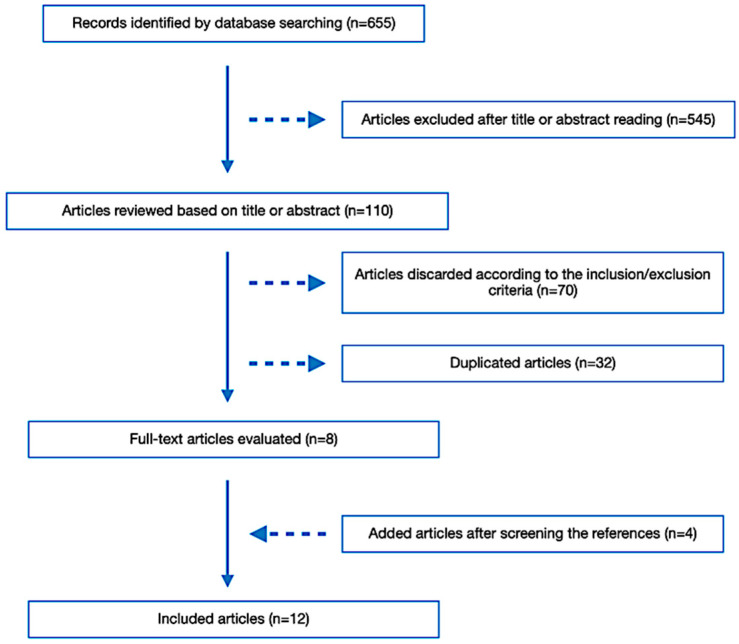
Flow chart of articles included in the review.

When the initial literature research was conducted up to 4 October 2024, 655 articles were identified. First, 32 duplicated articles were discarded. Afterward, by reviewing the titles and abstracts, 545 articles were also excluded. Then, 70 more articles were eliminated during the full-text review based on the inclusion and exclusion criteria. Screening the bibliography of the already selected ones, four additional articles were selected. As a result, 12 studies were finally included in the narrative review. The literature retrieval flow diagram is presented in Figure 1.

### 3.2. Preterm Birth

Maternal smoking in twin pregnancies has been significantly associated with an increased risk of preterm birth in most of the studies reviewed. 

Pollack et al. found that the risk of birth before 33 weeks in twin pregnancies was 1.84 times higher in smoking mothers compared to non-smokers (*p* < 0.05) [12]. The study reported that 42% of smoking pregnant women had a preterm birth, compared to only 31% of non-smokers (RR = 1.84, 95% CI: 1.25–2.71) [12].

Similarly, in the study by Schwendemann et al., it was reported that women who smoked during a twin pregnancy had a 1.35 times higher risk of delivering before 37 weeks compared to non-smokers (RR = 1.35, 95% CI: 1.15–1.58, *p* < 0.01) [13]. Additionally, among women who gained less than 0.5 kg per week during pregnancy, the risk of preterm birth increased significantly when combined with smoking (OR = 2.11, 95% CI: 1.45–3.07, *p* < 0.001) [13]. 

In the same way, Wisborg et al. found that smoking was significantly associated with an average reduction of 5 days in the gestational duration of twin pregnancies in smoking women compared to non-smokers (*p* < 0.01) [14]. Moreover, women who smoked between one and nine cigarettes per day had an average gestational age of 257 days (SD = 5.6), while those who smoked ten or more cigarettes per day had an average gestational age of 255 days (SD = 5.9) and non-smokers had an average of 263 days (SD = 5.2) [14]. Thus, a proportional reduction in gestational length was observed based on the number of cigarettes smoked [14].

Greatholder et al. also highlighted smoking as a critical risk factor for preterm birth in twin pregnancies [15]. In their study, 30% of smoking mothers gave birth before 34 weeks compared to 20% of non-smokers (*p* < 0.05) [15].

Additionally, Lucovnik et al., in their analysis of twin pregnancies complicated by preeclampsia, observed that women with a high BMI who were also smokers had an elevated risk of delivery before 37 weeks (OR = 1.92, 95% CI: 1.40–2.65, *p* < 0.01) [16]. This study found, consequently, a significant relationship between smoking, preeclampsia, BMI, and preterm birth [16].

On the other hand, Huisman et al. found significant hemodynamic alterations in smoking twin-pregnant women, with a 15% reduction in umbilical blood flow compared to non-smokers (*p* < 0.05), which could contribute to a higher risk of preterm birth [17]. Although this study does not report exact preterm birth rates, it suggests that the effect of smoking on hemodynamics may play a key role in pregnancy duration [17].

However, Martin et al. did not find a significant relationship between smoking and preterm birth in multiparous women (*p* = 0.21) [18]. In this study, smoking women did not have a higher rate of preterm birth compared to non-smokers, suggesting that the impact of smoking may vary depending on parity [18].

### 3.3. Fetal Growth Restriction

Smoking during twin pregnancies has also been linked to an increased risk of FGR, with multiple studies showing consistent data in this area. 

Schwendemann et al. and Inde et al. indicated that smoking twin-pregnant women had between 1.95 (95% CI: 1.51–2.52, *p* < 0.001) and 3.25 times greater risk of having small for gestational age (SGA) newborns compared to non-smoking mothers, respectively [13,19]. In their study, 28% of twins born to smoking mothers were classified as SGA compared to 15% of those born to non-smoking mothers [13]. This risk was higher in women who smoked more than ten cigarettes per day and had insufficient weight gain [13]. 

In the same way, Wisborg et al. noted that mothers who smoked more than ten cigarettes per day had twins with an average weight of 2550 g (SD = 450 g), compared to 2650 g (SD = 430 g) in non-smoking mothers with a *p*-value of <0.05, showing a significant relationship between smoking and fetal weight [14].

Similarly, in the study by Pollack et al., the neonates of smoking twin-pregnant women weighed on average 182 g less than those of non-smoking mothers, indicating a direct impact of smoking tobacco on the birth weight of twins (mean = 2450 g vs. 2632 g, *p* < 0.01) [12]. This finding was supported by Marleen et al., who reported that smoking increased the risk of FGR by 25% in twin pregnancies, with an average weight reduction of 200 g in neonates of smoking mothers (*p* < 0.05) [20]. Likewise, Salihu et al. observed a difference of 254 g in the average weight of neonates born to smoking mothers (95% CI: 200–308 g, *p* < 0.001) [21]. In their study, 35% of twins born to smoking mothers experienced FGR, compared to only 22% in twins of non-smoking mothers [21].

Furthermore, Whittaker et al. found that maternal smoking increased the risk of having an SGA baby by 33% in twins compared to singleton pregnancies, with a rate of 28% of SGA in smokers versus 15% in non-smokers [22].

On the other hand, Krotz et al. highlighted that the risk of FGR in twin pregnancies was higher when mothers had hypertension, which increased the likelihood of low birth weight, especially in smoking women (RR = 1.88, 95% CI: 1.20–2.94, *p* < 0.01) [23].

In addition, Lucovnik et al. noted that women with a high BMI (BMI > 30) who also smoked had a greater risk of FGR (OR = 2.22, 95% CI: 1.53–3.21, *p* < 0.001), with a 40% rate of low-birth-weight twins in this group [16].

However, Martin et al. reported that the effect of smoking on fetal growth was not as pronounced in multiparous women [18]. In their study, 19% of smoking mothers had twins with growth restriction, compared to 13% in non-smokers, although this difference was not statistically significant [18]. This suggests that other factors, such as parity, may mitigate the impact of smoking on fetal growth [18].

### 3.4. Preterm Premature Rupture of Membranes

PPROM is a major complication in twin pregnancies, and its relationship with maternal smoking has been less explored in the literature. 

Lucovnik et al. observed a higher incidence of PPROM (12%) in smoking twin-pregnant women with a high BMI and preeclampsia (*p* = 0.04) [16]. However, no statistically significant difference was found compared to non-smokers in absolute terms, only in combination with the risk factors mentioned before [16].

Similarly, the article by Whittaker et al. highlights that PPROM is a high-risk factor in twin pregnancies, particularly in smoking mothers. However, the study also did not find a statistically significant relationship between smoking and PPROM (RR = 1.05, 95% CI: 0.97–1.15, *p* > 0.05) [22]. 

On the other hand, Greatholder et al. reported that smoking twin-pregnant women had a higher incidence of PPROM, with an OR of 2.05 (95% CI: 1.45–2.91, *p* < 0.001). The study suggested that smoking influences the fragility of fetal membranes [15].

In contrast, Martin et al. did not find a clear relationship between smoking and PPROM in their analysis of twin pregnancies (*p* = 0.45) [18]. In their study, the rate of PPROM was similar between smokers and non-smokers, suggesting that other factors may be more implicated in this complication [18].

### 3.5. Fetal Death

Fetal death is one of the most severe complications associated with maternal smoking in twin pregnancies. Its association with maternal smoking in twin pregnancies is an unexplored field. 

Salihu et al. found that twin-pregnant women who smoked >10 cigarettes/day had a fetal death incidence of 1.2%, compared to 0.5% in non-smokers (*p* < 0.01) [21]. Additionally, the study showed a direct correlation between the number of cigarettes smoked and the risk of fetal death: mothers who smoked more than 20 cigarettes per day had a fetal death rate that rose to 2.1% (OR = 2.35, 95% CI: 1.56–3.54) [21].

However, Martin et al. did not find significant differences in the fetal death rate between smokers and non-smokers (*p* = 0.41) [18]. In their study, fetal mortality was similar in both groups, suggesting that smoking may not be a determining factor in all cases of fetal death but instead could be indirectly linked to other factors influencing fetal mortality, such as preterm birth and low birth weight [18].

### 3.6. Preeclampsia

Preeclampsia is a significant complication in twin pregnancies. Its relationship with maternal smoking has been explored in several studies. The data from the reviewed articles present mixed findings regarding the association of smoking tobacco with preeclampsia in twin pregnancies.

Lučovnik et al. showed that smoking was not significantly associated with preeclampsia in twin pregnancies (OR = 1.02, 95% CI: 0.58–1.81, *p* > 0.05) [16]. Instead, other factors such as high pre-pregnancy BMI and gestational diabetes were identified as more significant contributors to the development of preeclampsia in twin pregnancies [16]. 

Similarly, Krotz et al. highlighted that smoking does not have a significant effect on the development of preeclampsia in twin pregnancies. Although the relative risk of preeclampsia in twin pregnancies compared to singleton pregnancies ranges from 2.8 to 4.4, smoking was not found to be a major modifying factor [23]. Other factors, such as parity and maternal age, were identified as more prominent contributors to preeclampsia risk in twin gestations [23].

However, Martin et al. observed a lower incidence of preeclampsia in multiparous twin pregnancies among smokers compared to non-smokers (16.6% vs. 18.6%, *p* = 0.04), suggesting a potential protective effect of smoking [18]. However, this effect was not significant for primiparous women [18]. 

### 3.7. Neonatal and Post-Natal Consequences

Smoking during twin pregnancies has been associated with various neonatal complications, including both respiratory problems that may become chronic and neurodevelopmental disorders, affecting the child’s well-being during childhood and adolescence.

Whittaker et al. noted that twins of smoking mothers had a higher rate of admission to the neonatal intensive care unit (NICU), with a 1.28 times higher risk of severe complications (RR = 1.28, 95% CI: 1.09–1.50, *p* < 0.01) [22]. These results are consistent with those of Lucovnik et al., who found that 35% of the twins of smoking mothers with preeclampsia required admission to the NICU, experienced recurrent respiratory complications, and had delays in neurological development within the first three years, compared to 15% of non-smoking mothers’ neonates (OR = 1.88, 95% CI: 1.20–2.94, *p* < 0.01) [16]. Similarly, Schwendemann et al. also found that 30% of twins of smoking mothers required NICU admission for respiratory complications, compared to 18% of twins of non-smoking mothers, which was also statistically significant (OR = 1.75, 95% CI: 1.25–2.46, *p* < 0.001) [13]. Specifically, this study showed that the risk of these problems was higher in neonates born to mothers who smoked more than ten cigarettes per day [13].

Along the same lines, Pollack et al. indicated that the neonates of smoking twin-pregnant women had an increased risk of requiring respiratory assistance at birth, especially those born weighing less than 2500 g [12]. Thus, 28% of the twins of smoking mothers in the study required some form of respiratory assistance in the delivery room, compared to 15% of the twins of non-smoking mothers (*p* < 0.01) [12].

On the other hand, Huisman et al. observed that smoking induced a 12% increase in the fetal heart rate of twins of smoking mothers (an average of 8 bpm higher in fetuses of smokers, *p* = 0.03), suggesting more significant fetal stress during pregnancy, predisposing to respiratory complications in the newborn [17]. Specifically, respiratory problems were recorded in 25% of neonates of smoking mothers in the first month of life, compared to 10% of those born to non-smoking mothers [17].

Furthermore, Schwendemann et al. found that twins of smoking mothers had a significantly higher risk of developing chronic respiratory problems in the first years of life [13]. Up to 25% of neonates exposed to tobacco in utero developed asthma or chronic bronchitis before the age of 5, compared to 10% of non-exposed neonates (*p* < 0.01), and by age 10, 28% of the twins exposed to tobacco continued requiring medication for asthma, compared to 12% of non-exposed (*p* < 0.01) [13]. There was also a higher rate of hospitalizations for respiratory infections in the first three years of life, with a rate of 18% in children of smoking mothers compared to 9% in non-exposed (OR = 2.0, 95% CI: 1.45–2.75, *p* < 0.001) [13]. These findings are consistent with those of Krotz et al., who also indicated that twins of mothers with hypertension and smoking had a higher risk of developing asthma and chronic bronchitis, with a 25% increase in the incidence of chronic respiratory problems (OR = 1.25, 95% CI: 1.10–1.45, *p* < 0.05) [23].

Regarding behavioral disorders, Salihu et al. showed that twins born to smoking mothers had a higher risk of experiencing learning delays and cognitive difficulties [21]. In total, 22% of the twins exposed to tobacco during pregnancy were diagnosed with learning problems before the age of 7, compared to 10% of non-exposed twins (OR = 2.25, 95% CI: 1.42–3.55, *p* < 0.01) [21]. Moreover, the study noted that these twins had a higher incidence of behavioral disorders and concentration difficulties [21]. This association is attributed to prenatal nicotine exposure, which interferes with neurological development, affecting executive function and attention circuits in the brain [24].

In this way, Pollack et al. similarly indicated that neonates who experienced FGR as a result of maternal smoking had slower physical and neurological development during childhood, with a delay of up to 15% in growth compared to their non-exposed peers, measured at 18 months of age (*p* < 0.05) [12].

However, Martin et al. did not find significant differences in the incidence of respiratory complications between the neonates of smoking and non-smoking mothers [18]. Nonetheless, the authors suggest that smoking could be related to other long-term developmental problems, such as neurological or cognitive delays, noting that twins born to smoking mothers have slower overall development during the early years of life [18]. Thus, 20% of these children exhibited delayed language development compared to 10% of non-exposed twins (*p* < 0.05), indicating a relationship between prenatal tobacco exposure and long-term cognitive delays [18].

## 4. Discussion

### 4.1. Preterm Birth

Preterm birth is a common complication in twin pregnancies, and maternal smoking has been shown to increase this risk significantly. 

The reviewed studies found that smoking mothers of twins had a higher risk of preterm delivery, both before 33 weeks [12], as well as at 34 weeks [14,15] and 37 weeks [13], showing a proportional reduction in gestational length with the number of cigarettes smoked per day [13].

Other risk factors that enhanced the association between smoking and preterm birth in twin pregnancies include preeclampsia and BMI [13,16]. Twin-pregnant women who smoked had preeclampsia, and a high BMI presented an elevated risk of delivery before 37 weeks [16], as well as insufficient weekly maternal weight gain (less than 0.5 kg per week), reporting that twin-pregnant women who smoked and gained low weight also had a higher risk of preterm delivery [13].

These results are comparable to those seen in singleton pregnancies, in which smoking has been linked to an increased risk of preterm birth due to nicotine’s vasoconstrictive effects that reduce uterine and placental blood flow, leading to fetal hypoxia [25,26].

This mechanism could also apply to twin pregnancies, where the greater oxygen demand from two fetuses may increase susceptibility to hypoxia. Additionally, the more complex placental development in multiple pregnancies could amplify the adverse effects of smoking by further compromising placental and umbilical circulation [17].

Moreover, smoking induces the release of prostaglandins, substances that can trigger uterine contractions and contribute to the early onset of labor. Goldenberg et al. (2008) demonstrated that smoking women had higher levels of prostaglandins E2 and F2-alpha, which are essential in the induction of labor [27]. This effect, already documented in singleton pregnancies, could be exacerbated in twin pregnancies due to the inherent higher risk of preterm labor in these cases.

In contrast, Martin et al. did not find a significant relationship between smoking and preterm birth in multiparous women [18]. This could be due to a reduced sensitivity to smoking in women who have experienced multiple pregnancies, as their circulatory system may have better adapted to the hemodynamic changes. Additionally, in multiparous women, other factors such as birth weight in previous pregnancies and weight gain during the current pregnancy may play a more critical role than smoking in the risk of preterm birth.

### 4.2. Fetal Growth Restriction

Fetal growth restriction is a common complication in twin pregnancies and has been linked to smoking in several studies. 

Most of the studies reviewed showed that smoking mothers had an increased risk of having SGA newborns, with a mean birth weight reduction of 100–254 g, depending on the study analyzed [12,13,14,20,21,22].

Other risk factors that amplified the effect of smoking on the development of FGR in twin pregnancies include hypertension [23] and a BMI > 30 [16].

In singleton pregnancies, smoking is also associated with an increased risk of fetal growth restriction, mainly due to the same mechanisms that increase the incidence of preterm birth. Specifically, the vasoconstrictive effects of nicotine cause a reduction in the diameter of spiral arteries in the placenta, thereby decreasing the blood flow and reducing the supply of nutrients and oxygen to the fetus, ultimately leading to intrauterine growth restriction [25,26].

In twin pregnancies, this effect would also be exacerbated due to the greater nutritional demands of two fetuses, causing competition for available resources [25,28].

Furthermore, Doppler studies have shown increased resistance in the umbilical arteries of fetuses of smoking mothers, further decreasing oxygen flow to the fetus and exacerbating fetal growth restriction in both singleton [28] and multiple pregnancies [17].

However, some studies, such as Martin et al., did not find a significant relationship between smoking and fetal growth restriction in multiparous women [18]. A possible explanation for these results is that, in multiparous women, as suggested for preterm birth, the uterus and placenta may have better adapted to the changes induced by smoking, thus mitigating some of its adverse effects [18]. Additionally, in some cases, multiparous women experience greater weight gain during pregnancy, which could counterbalance the adverse effects of smoking on fetal growth [18].

### 4.3. Preterm Premature Rupture of Membranes

PPROM is a common complication in twin pregnancies, often leading to preterm birth and associated neonatal morbidity. Some evidence suggests a potential link between maternal smoking and an increased risk of PPROM, although the current evidence is limited, and the findings remain inconsistent and inconclusive.

For instance, while some studies found that smoking mothers had a higher incidence of PPROM, the results were not statistically significant [16,22]. This inconsistency may stem from variability in study designs, differences in the number of cigarettes participants smoke, or the confounding influence of other risk factors.

The biological mechanism underlying PPROM has been extensively studied in singleton pregnancies and is related to oxidative damage induced by tobacco smoke in the amniotic membranes. The toxic components of cigarette smoke, such as carbon monoxide and free radicals, can weaken the structure of the membranes by reducing collagen production, thinning them and thus making them more susceptible to rupture [29].

This mechanism could be extrapolated to twin pregnancies but with an increased risk due to the greater intrauterine pressure exerted by two fetuses on the membranes, making them more prone to premature rupture [27].

However, conflicting evidence exists, as some studies like that of Martin et al. did not find a significant relationship between smoking and PPROM. This could be explained by the variability in the number of cigarettes smoked by study participants or the influence of other factors, such as intrauterine infections, a well-documented cause of PPROM, that may act as a more robust determinant in some instances [27]. Furthermore, conditions such as polyhydramnios or discordant growth in twin pregnancies could exert mechanical stress on the membranes, playing a more critical role than smoking in the etiology of PPROM [27].

These mixed findings underscore the complexity of PPROM and highlight the need for further research to disentangle the interplay between maternal smoking and other contributory factors. Examining the synergistic impact of smoking alongside other conditions, such as infections or polyhydramnios, could provide a more nuanced understanding of the mechanisms leading to PPROM in twin pregnancies.

### 4.4. Fetal Death

The risk of fetal death is higher in twin pregnancies due to complications inherent to this type of gestation, such as unequal intrauterine growth and twin-to-twin transfusion syndrome. This means that the adverse effects of smoking may be more pronounced and further increase this risk. However, the evidence in this area remains limited, with only a few studies analyzing this specific outcome.

An increased rate of fetal death was reported in smoking twin-pregnant women, ranging from 1.2% to 2.1%, depending on the number of cigarettes smoked per day [21]. 

These results are consistent with findings in singleton pregnancies, where smoking has also been identified as a significant risk factor for fetal mortality. Cnattingius et al. observed that the risk of fetal death doubles in women who smoke more than ten cigarettes per day, mainly due to placental insufficiency and chronic hypoxia affecting the fetus [30]. This not only results in compromised fetal growth, as mentioned earlier, but in some cases, can lead to fetal death [30].

In twin pregnancies, the risk of fetal death due to smoking is particularly high due to the increased physiological burden on the placenta to nourish two fetuses. Friedman and Polifka noted that the placenta in multiple pregnancies has a limited capacity to compensate for the damage caused by smoking, which increases the risk of placental insufficiency [26]. This impaired placental blood flow, exacerbated by smoking, reduces the oxygen supply to both fetuses, which is a crucial factor contributing to fetal death [26].

However, some studies, such as that of Martin et al., did not find significant differences in fetal death rates between smokers and non-smokers [18]. This could be due to the small sample of fetal death cases in their cohort or the influence of additional factors, such as advanced prenatal care, which may have mitigated some of the adverse effects of smoking in their study population. 

Therefore, it is essential to highlight that fetal death is a multifactorial outcome. While smoking is an important factor, it is not always the primary determinant but an aggravating factor that exacerbates the effects of other complications. Further research is needed to clarify the precise role of smoking in fetal death in the context of twin pregnancies, where multiple variables interact to influence outcomes.

### 4.5. Preeclampsia

Studies report that the risk of preeclampsia is approximately four times higher in twin pregnancies compared to singleton pregnancies [23,31]. This risk is mainly compounded by factors such as nulliparity, higher maternal age, high pre-pregnancy BMI, and gestational diabetes. Still, smoking appears to have little effect on twin pregnancies, with no significant results [16,23]. Only one study observed a lower incidence of preeclampsia in smoking twin pregnancies in the specific subgroup of multiparous women [18].

In contrast, in singleton pregnancies, smoking has been shown to have a protective effect in all groups, reducing the risk of preeclampsia [32,33]. This has led to the hypothesis that smoking may influence the pathophysiology of preeclampsia by reducing placental blood flow and oxygen delivery, which might trigger compensatory mechanisms that mitigate some of the characteristics of preeclampsia, such as hypertension [32,33].

The inconsistency in smoking’s effect between singleton and twin pregnancies may be due to the greater complexity of twin pregnancies. Factors such as higher placental mass and increased strain on maternal systems likely diminish any potential impact that smoking might have in preventing preeclampsia [16,23]. Additionally, the physiological adaptations required to sustain two fetuses may alter the way the maternal body responds to smoking, potentially offsetting any benefits that might be seen in singleton pregnancies [16].

Furthermore, it is essential to consider that preeclampsia is a multifactorial condition. While smoking may play a role in modifying the risk, other factors, such as maternal health, placental function, and genetic predispositions, are likely to interact in complex ways to determine the outcome. Therefore, further studies focusing specifically on the interactions between smoking and other risk factors in twin pregnancies are necessary to fully understand how smoking influences the development of preeclampsia in this high-risk population.

### 4.6. Neonatal and Post-Natal Complications

The neonatal consequences of smoking in twin pregnancies are particularly severe and, in many cases, may become chronic over time, significantly affecting the quality of life, health, and long-term development of the newborns.

Most of the reviewed studies found that neonates born to smoking mothers had a higher risk of respiratory complications compared to those born to non-smoking mothers, more frequently requiring respiratory assistance and even admission to the NICU [12,13,16,17,22]. Moreover, the risk of these problems was higher in neonates born to mothers who smoked a greater number of cigarettes per day [13].

These findings are consistent with studies in singleton pregnancies that have demonstrated that maternal smoking interferes with fetal lung development, predisposing newborns to respiratory problems at birth, such as respiratory distress syndrome [34]. Additionally, maternal smoking is also associated with increased levels of carboxyhemoglobin in the fetus, which reduces the fetus’s ability to transport oxygen and may further compromise lung development.

In twin pregnancies, where the oxygen exchange between the mother and two fetuses is already compromised due to the greater physiological demands, this effect is amplified, resulting in a higher incidence of neonatal respiratory complications [12,13,16,17,22].

On the other hand, this review found that 25% of twins exposed to tobacco in utero developed asthma or chronic bronchitis over time [13,23].

In this line, Jaakkola and Gissler (2004) documented similar results in singleton pregnancies, mainly explained by prenatal exposure to carbon monoxide and other toxic compounds from tobacco smoke, which interfere with normal fetal lung development [34].

In twin pregnancies, this effect may be even more pronounced due to the combination of frequent complications, such as low birth weight and preterm birth, that, on their own, already increase the risk of long-term respiratory problems, such as respiratory distress syndrome and chronic lung disease. When combined with the effects of maternal smoking, these inherent complications of twin pregnancies lead to a much higher cumulative risk of chronic respiratory issues during development.

Regarding the neurological and cognitive development of children, this review found that twins exposed to maternal smoking showed a higher incidence of behavioral issues and attention difficulties [12,21], representing a doubled risk [21]. Additionally, not only was a neurodevelopmental delay observed, but also physical developmental postponement, with a growth delay of up to 15% in twins who experienced FGR as a result of maternal smoking [12].

Similarly, many studies found that in singleton pregnancies, children of smoking mothers have a higher risk of experiencing cognitive problems and learning difficulties throughout their school years [35,36,37]. Prenatal exposure to tobacco smoke can alter brain development, affecting synapse formation and myelination of neurons [35,36]. This can lead to language development difficulties, memory problems, and a reduced attention span in children [35,36]. Additional studies have found a link between maternal smoking and ADHD, suggesting that prenatal exposure to tobacco smoke may interfere with the neurological circuits responsible for regulating behavior and attention [36,37,38].

In twin pregnancies, neurological and cognitive problems may be more severe due to the higher likelihood of preterm birth, a condition already associated with an increased risk of neurological development problems [39]. Studies have shown that preterm babies are at greater risk of damage to the white matter of the brain, leading to delays in motor and cognitive development. When combined with the effects of maternal smoking, the risk of developmental delays is even higher [39].

Maternal smoking has been linked in singleton pregnancies to increased levels of oxidative stress and low-grade chronic inflammation in the fetus, which can have long-term consequences on the child’s neurological and physical development. Oxidative stress affects the body’s ability to repair cellular damage, which can interfere with the proper development of organs, including the lungs and brain [24]. This explains why children exposed to maternal smoking have a higher risk of developing not only respiratory and cognitive problems but also metabolic and cardiovascular diseases in adulthood.

Moreover, studies such as that of Ernst et al. suggest that the effects of maternal smoking may be transgenerational [40]. This means that children of mothers who smoked during pregnancy may not only experience health problems throughout their lives but could also pass these risks on to their own children [40]. This transmission of risks across generations is due to epigenetic changes induced by prenatal exposure to tobacco smoke, which affects the expression of genes responsible for respiratory, metabolic, and neurological health [40].

Finally, it is essential to remark that socioeconomic factors play a crucial role in the prevalence and continuation of smoking during pregnancy. A study conducted in Romania reported that women with low income and unemployment were more likely to continue smoking throughout pregnancy, despite understanding its associated risks. Although this study did not specifically focus on twin pregnancies, its findings emphasize the need to address social inequalities in smoking cessation strategies, particularly in high-risk pregnancies like twin gestations. These results highlight the importance of tailored public health interventions aimed at vulnerable populations, considering how socioeconomic disparities amplify the risks of adverse obstetric and neonatal outcomes associated with maternal smoking [41].

## 5. Limitations and Future Research

It is important to note that the majority of the studies included in this review rely on self-reported data to assess maternal smoking during pregnancy, while a smaller proportion do not specify the source of this information. This reliance on self-reported data introduces potential limitations, as it may be subject to recall bias or underreporting due to the social stigma associated with smoking during pregnancy. These methodological weaknesses should be carefully considered when interpreting the findings, highlighting the need for future research employing objective measures, such as biochemical markers, to improve accuracy and reliability.

Furthermore, there is considerable variability in the methodologies employed by the studies included in this review. Differences in study designs, data collection methods, and population characteristics may affect the comparability of results and pose challenges for their interpretation. This heterogeneity underscores the need for standardized protocols in future research to enable more consistent and reliable study comparisons.

Additionally, only a few studies provide detailed subgroup analyses by the number of cigarettes smoked daily, limiting the ability to explore dose-response effects. This lack of granularity in data analysis constrains our understanding of how varying levels of smoking exposure impact obstetric and neonatal outcomes in twin pregnancies.

Moreover, the majority of studies focus exclusively on active smoking, with limited or no consideration of passive smoking or combined exposure. This narrow focus represents an essential gap in the literature and may restrict the generalizability of findings to populations exposed to secondhand smoke during pregnancy. Comprehensive assessments of both active and passive smoking exposures are needed to provide a more holistic understanding of the risks associated with maternal smoking.

Finally, while factors such as diet, maternal health, physical activity, and exposure to environmental pollutants could significantly influence pregnancy outcomes in the context of maternal smoking, they are largely underexplored in the included studies. Only a limited number of studies briefly mention these factors, and none provide a comprehensive analysis of their interactions with smoking. This gap in the literature limits the ability to assess the multifactorial nature of smoking-related twin pregnancy outcomes and highlights the importance of future research addressing these variables.

## 6. Conclusions

This narrative review shows that maternal smoking in twin pregnancies is significantly associated with an increased risk of perinatal complications, including preterm birth and fetal growth restriction. These results were more evident when smoking was associated with additional risk factors, suggesting that smoking may not be the only factor responsible and that other factors could moderate its impact. However, an association with preeclampsia was not found. Regarding PPROM and fetal death, there remain areas with mixed or inconsistent results in the current literature, and further research is needed to clarify the relationship.

As for post-natal complications, the children of smoking mothers have an increased risk of respiratory problems, such as asthma and chronic bronchitis, which can persist throughout life. Moreover, there is a higher incidence of neurological and cognitive developmental disorders, as well as delays in physical and mental development. These adverse effects of smoking affect neonates not only in the short term but also have long-term implications for their health and development.

All these complications are primarily due to reduced oxygen supply to the fetus, caused by nicotine-induced vasoconstriction and carbon monoxide binding to hemoglobin, leading to placental insufficiency and fetal hypoxia. Additionally, smoking triggers oxidative stress and inflammation, which damage placental function and impair fetal normal development. In multiple pregnancies, where the physiological burden is greater, these adverse effects seem even more pronounced than in singletons.

In conclusion, maternal smoking in twin pregnancies not only increases the risk of obstetric complications but also has lasting consequences for the health and development of the children. It is crucial to promote effective smoking cessation strategies during pregnancy, especially in the case of multiple gestations and vulnerable populations, to reduce these risks. Education and close monitoring of pregnant women who smoke should be a priority in prenatal care to improve both maternal and neonatal outcomes.

## Figures and Tables

**Table 1:** Characteristics, results, and conclusions of the studies included in the review.

Studies Included	First Author and Year	Type of Study	Participants	Smoking Data	Results	Conclusions
[12]	Pollack, H. (2000)	Systematic review (23 studies from the USA)	n = 3,899,589 births including singletons and twins (96,785). Active smokers.	Self reported Number of cigarettes smoked not detailed	- Women who smoked had a significantly higher risk of preterm birth (RR = 1.84) and low birth weight (*p* < 0.01). - Twins born to smoking mothers weighed 182 g less on average than those born to non-smokers. - Neonates affected by FGR due to maternal smoking showed delayed physical and neurological development, with up to a 15% slower growth compared to non-exposed peers by 18 months of age (*p* < 0.05).	- Maternal smoking in twin pregnancies significantly increases the risk of preterm birth, low birth weight, and delayed physical and neurological development. - The study underscores that smoking exacerbates risks in twins more than in singletons, highlighting the need for targeted smoking cessation programs for pregnant women.
[13]	Schwendemann, W. (2005)	Multicenter retrospective cohort study (USA)	n = 11,827 twins Not specified if active or passive smokers.	Not specified Number of cigarettes smoked not detailed	- Maternal smoking was associated with an increased risk of fetal growth restriction (OR = 1.95) and preterm birth (RR = 1.35). - This risk was higher among mothers who smoked more than 10 cigarettes per day and gained less than 0.5 kg per week. - 30% of twins born to smoking mothers required NICU admission for respiratory complications, compared to 18% of non-smoking mothers’ twins (OR = 1.75, 95% CI: 1.25–2.46, *p* < 0.001), with a higher risk in mothers smoking more than 10 cigarettes per day. - Up to 25% of neonates exposed to tobacco in utero developed asthma or chronic bronchitis before age 5, compared to 10% of non-exposed neonates (*p* < 0.01). By age 10, 28% of exposed twins still required asthma medication, compared to 12% of non-exposed (*p* < 0.01). - Twins of smoking mothers had a higher rate of hospitalizations for respiratory infections in the first 3 years of life (18%) compared to non-exposed children (9%) (OR = 2.0, 95% CI: 1.45–2.75, *p* < 0.001).	- Maternal smoking significantly increases the risk of fetal growth restriction and preterm birth in twin pregnancies. The risk is higher among mothers who smoke more than 10 cigarettes per day and gain less than 0.5 kg per week. - The study emphasizes the importance of smoking cessation and adequate maternal weight gain during pregnancy to mitigate adverse outcomes.
[14]	Wisborg, K. (2001)	Prospective cohort study (Denmark)	n = 401 twin pregnancies. Active smokers.	Self reported Participants divided in three groups depending on the number of cigarettes smoked (1–9, 10–19, and 20+).	- Twin-pregnant women who smoked had an average reduction of 5 days in gestational length (*p* < 0.01). - Women who smoked more than 10 cigarettes per day had an average gestational age of 255 days, compared to 263 days for non-smokers. - Mothers who smoked more than 10 cigarettes per day had twins with an average weight of 2550 g (SD = 450 g), compared to 2650 g (SD = 430 g) in non-smokers (*p* < 0.05).	- Maternal smoking in twin pregnancies is linked to a significant reduction in gestational length and birth weight, especially for mothers smoking more than 10 cigarettes per day. -The study underscores the importance of smoking cessation to reduce risks associated with compromised neonatal health.
[15]	Greatholder, I. (2023)	Prospective observational study (UK)	n = 277 twin pregnancies. Not specified if active or passive smokers	Self reported Number of cigarettes smoked not detailed	- 30% of smoking mothers gave birth before 34 weeks, compared to 20% of non-smokers (*p* < 0.05). - Smoking twin-pregnant women had a higher incidence of PPROM, with an OR of 2.05 (95% CI: 1.45–2.91, *p* < 0.001).	- Maternal smoking in twin pregnancies is associated with higher rates of preterm birth (before 34 weeks) and increased incidence of PPROM.
[16]	Lučovnik, M. (2012)	Case-control study (Slovenia)	n = 3885 twins (181 with preeclampsia and 542 controls). Not specified if active or passive smokers.	Not specified Number of cigarettes smoked not detailed.	- No significant association between smoking and preeclampsia was found (OR = 1.02). - Twin-pregnant smoker women with preeclampsia and a high BMI had an elevated risk of delivery before 37 weeks (OR = 1.92, 95% CI: 1.40–2.65, *p* < 0.01). - Women with a BMI > 30 who also smoked had a greater risk of FGR (OR = 2.22, 95% CI: 1.53–3.21, *p* < 0.001), with a 40% rate of low-birth-weight twins in this group. - A higher incidence of PPROM (12%) in smoking twin-pregnant women with a high BMI and preeclampsia (*p* = 0.04). - 35% of twins born to smoking mothers with preeclampsia required NICU admission, experienced recurrent respiratory complications, and had delays in neurological development within the first 3 years, compared to 15% of neonates from non-smoking mothers (OR = 1.88, 95% CI: 1.20–2.94, *p* < 0.01).	- Maternal smoking in twin pregnancies was not significantly associated with preeclampsia (OR = 1.02). However, smoking combined with high BMI increased risks for adverse outcomes, including preterm birth and fetal growth restriction (FGR). Moreover, smoking mothers with preeclampsia had higher rates of PPROM and NICU admissions for their twins, who also experienced more respiratory issues and developmental delays. - The study highlights the compounded risks when smoking coincides with other factors like high BMI, emphasizing targeted interventions for high-risk twin pregnancies.
[17]	Huisman, M. (1997)	Experimental study (International)	n = 10 early twin pregnancies. Active smokers.	Not specified All participants smoked > 5 cigarettes per day.	- Nicotine consumption caused a 15% reduction in umbilical blood flow (*p* < 0.05) and increased fetal heart rate, contributing to a higher risk of preterm birth. - 25% of neonates of smoking mothers has respiratory problems in the first month of life, compared to 10% of those born to non-smoking mothers.	- Nicotine consumption during early twin pregnancies significantly affects fetal hemodynamics, causing a 15% reduction in umbilical blood flow and increased fetal heart rate, contributing to a higher risk of preterm birth. Additionally, neonates of smoking mothers had a higher incidence of respiratory issues in the first month of life. - These findings underscore the detrimental impact of nicotine on fetal development and early neonatal health in twin pregnancies.
[18]	Martin, C. (2000)	Retrospective cohort study (Scotland)	n = 1575 twins (born between 1969 and 1997). Active smokers.	Self reported Participants divided in three groups depending on the number of cigarettes smoked (1–9, 10–19, and 20+).	- A significantly lower incidence of preeclampsia was observed in multiparous smoking women (*p* = 0.04). - A significant relationship between smoking and preterm birth in multiparous women was not found (*p* = 0.21) - 19% of smoking mothers had twins with growth restriction, compared to 13% in non-smokers, although this difference was not statistically significant. - The rate of PPROM was similar between smokers and non-smokers (*p* = 0.45). - Perinatal mortality rates were similar between smokers and non-smokers (49.3 per 1000 in smokers vs. 47.4 in non-smokers). - The late miscarriage rate was significantly higher in smokers (54.1 per 1000) compared to non-smokers (35.0 per 1000, *p* = 0.012). - There were no significant differences in the incidence of respiratory complications between the neonates born to smokers and non-smokers. - 20% of twins exhibited delayed language development among other developmental problems compared to 10% of non-exposed twins (*p* <0.05).	- Maternal smoking in twin pregnancies was linked to a lower incidence of preeclampsia in multiparous women but higher late miscarriage rates and developmental delays in exposed twins. No significant association with preterm birth or growth restriction was found. - These findings highlight the risks associated with smoking during twin pregnancies, despite some observations of reduced preeclampsia rates.
[19]	Inde, Y. (2011)	Retrospective cohort study (Japan)	n = 340 twin Japanese dichorionic twin pregnancies, of whom 126 (37%) had at least one SGA newborn. Not specified if active or passive smokers	Not specified Number of cigarettes smoked not detailed	- Maternal smoking (OR 3.25), pregnancy-induced hypertension (OR 2.00), and low maternal weight gain were significant risk factors for SGA in dichorionic twins.	- Maternal smoking, pregnancy-induced hypertension and low maternal weight gain are significant risk factors for SGA in dichorionic twin pregnancies. - The study highlights that smoking is a modifiable factor that can be managed to reduce the risk of adverse outcomes, emphasizing the importance of targeted interventions for maternal health to improve perinatal outcomes.
[20]	Marleen, S. (2018)	Systematic review (59 studies from various countries)	n = 2,930,958 twin pregnancies Not specified if active or passive smokers	Not specified Number of cigarettes smoked not detailed	- Smoking increased the risk of FGR by 25% in twin pregnancies, with an average weight reduction of 200 g in neonates of smoking mothers (*p* < 0.05).	- Maternal smoking in twin pregnancies increased the risk of fetal growth restriction (FGR). - This highlights the significant impact of smoking on fetal development and underscores the importance of smoking cessation to improve pregnancy outcomes.
[21]	Salihu, H. (2005)	Retrospective cohort study (USA)	n = 163,901 twins born alive between 1995 and 1998, of which 19,234 mothers were smokers (11.7%). Active smokers.	Self reported Number of cigarettes smoked not detailed	- A difference of 254 g in the average weight of neonates born to smoking mothers was reported (95% CI: 200–308 g, *p* < 0.001). - 35% of twins born to smoking mothers experienced FGR, compared to only 22% in twins of non-smoking mothers. - A higher risk of fetal mortality was also observed (1.2% in smokers vs. 0.5% in non-smokers, *p* < 0.01), which increased up to 2.1% following a direct correlation depending on the number of cigarettes smoked - 22% of twins exposed to tobacco during pregnancy were diagnosed with learning problems before the age of 7, compared to 10% of non-exposed twins (OR = 2.25, 95% CI: 1.42–3.55, *p* < 0.01) and behavioral disorders.	- Maternal smoking in twin pregnancies is associated with lower birth weights, higher rates of fetal growth restriction, increased fetal mortality, and long-term learning and behavioral issues. - These findings highlight the critical need for smoking cessation during pregnancy to improve neonatal and developmental outcomes.
[22]	Whittaker, M. (2023)	Narrative review (UK)	n = number of studies and sum of participants not reported in the review. Not specified if active or passive smokers	Not specified Number of cigarettes smoked not detailed	- Smoking was a relevant factor for NICU admission due to respiratory issues (RR = 1.28, *p* < 0.01). - Maternal smoking increased the risk of having an SGA baby by 33% in twins compared to singleton pregnancies, with a rate of 28% of SGA in smokers versus 15% in non-smokers. - PPROM is a high-risk factor in twin pregnancies, particularly in smoking mothers, although a statistically significant relationship was not found (RR = 1.05, 95% CI: 0.97–1.15, *p* > 0.05).	- Maternal smoking in twin pregnancies significantly increases the risk of NICU admission due to respiratory issues and raises the incidence of SGA babies. Although smoking mothers had a higher risk of PPROM, the relationship was not statistically significant. - These findings emphasize the importance of smoking cessation to reduce adverse neonatal outcomes in twin pregnancies.
[23]	Krotz, S. (2002)	Systematic review (23 studies from USA, Israel, UK, Norway, Qatar, and Canada)	Review of 23 studies of twin pregnancies. Not specified if active or passive smokers	Not specified Number of cigarettes smoked not detailed	- A higher risk of preeclampsia (RR 2.8–4.4) was found in twin pregnancies, but no significant relationship was observed between smoking and preeclampsia. - The risk of FGR in twin pregnancies was higher when mothers had hypertension, which increased the likelihood of low birth weight, especially in smoking women (RR = 1.88, 95% CI: 1.20–2.94, *p* < 0.01) - Twins of mothers with hypertension and smoking had a higher risk of developing asthma and chronic bronchitis, with a 25% increase in the incidence of chronic respiratory problems (OR = 1.25, 95% CI: 1.10–1.45, *p* < 0.05).	- Twin pregnancies carry a higher risk of preeclampsia, but maternal smoking was not significantly related to preeclampsia itself. However, smoking combined with hypertension increased the risk of fetal growth restriction (FGR) and chronic respiratory problems in twins. - This highlights the compounded impact of smoking and maternal hypertension on adverse pregnancy and neonatal outcomes in the third trimester of pregnancy, with their effectiveness, safety, and immune response comparable to that of the general population.

## Data Availability

The authors can provide additional information upon request.
